# COVID-19: sitting is the new smoking; the role of exercise in augmenting the immune system among the elderly

**DOI:** 10.4314/ahs.v21i1.25

**Published:** 2021-03

**Authors:** Olorunfemi Tokunbo, Taiwo Abayomi, Damilare Adekomi, Ibukun Oyeyipo

**Affiliations:** 1 Department of Anatomy, Faculty of Basic Medical Sciences, College of Health Sciences, Osun State University, PMB 4494, Osogbo, Osun State, Nigeria; 2 Department of Physiology, Faculty of Basic Medical Sciences, College of Health Sciences, Osun State University, PMB 4494, Osogbo, Osun State, Nigeria

**Keywords:** Exercise, immune system, sedentary lifestyle, myokines

## Abstract

**Introduction:**

Like smoking, sedentary lifestyle is an issue of great concern because of its deleterious health challenges and implications. Given the global spread of the new coronavirus (COVID-19), social isolation regulations and laws have been implemented in many countries to contain the spread of the virus and this has caused a drastic shift from the usual physically demanding life to a sedentary lifestyle characterized by significantly reduced physical activities and prolong sitting.

**Methods/Data Source:**

Human and nonhuman primate literature was examined to compare experimental and clinical modulation of inflammatory cytokines by exercised-induced myokines.

**Data synthesis:**

Experimental and clinical evidence was used to examine whether exercised-induced myokines can prime the immune system of the elderly population during the COVID-19 pandemic.

**Conclusion:**

The immune system changes with advancement in age which increases the likelihood of infectious disease morbidity and mortality in older adults. Several epidemiological studies have also shown that physical inactivity among geriatric population impacts negatively on the immune system. Evidences on the importance of exercise in priming the immune system of elderly individuals could be an effective therapeutic strategy in combating the virus as it may well be a case of “let those with the best immune system win”.

## Introduction

The World Health Organization (WHO) used the term 2019 novel coronavirus (COVID-19) to refer to a coronavirus that affected the respiratory tract of patients with pneumonia in Wuhan, China on 29^th^ December 2019. What started as an epidemic later escalated rapidly into a pandemic and cases have been reported globally with over 8.8 million confirmed cases and 465,000 COVID-19 associated deaths as of 21^st^ June, 20201. The African continent is not left out of this global disease burden with 297,112 confirmed cases and 7,191 deaths according to the Africa center for disease control (ACDC). In Nigeria, more than 19,000 reported cases have been reported with over 480 deaths as at the time of writing this article.

The virus is generally associated with upper respiratory tract infections, with common signs and symptoms which include fever, cough and shortness of breath among others^2^. In more severe cases, infection can cause pneumonia, severe acute respiratory syndrome and sometimes death^3^. The likelihood of hospitalization, severe illness and death increases in persons over 65 years of age and those with underlying risk factors including hypertension, diabetes, cardiovascular disease, chronic respiratory disease, cancer, compromised immune status, and obesity^1^,^2^. A strong immune response is required to eliminate the virus and to stop disease progression to severe stages during the incubation and non-severe stages. For the development of an endogenous protective immune response to the virus at the early stages of infection, the host should be in good general health and have a good genetic background that elicits specific antiviral immunity^3^,^4^. Therefore, strategies to boost immune responses, especially in the elderly population are certainly important.

Given the spread of the new coronavirus and its impacts on human health, countries around the world are implementing measures to specifically inhibit its spread. From national quarantines to school closures, most nations of the world are under some form of restriction or the other. While lockdown isn't a technical term used by public health officials, it can refer to anything from mandatory geographic quarantines to non-mandatory medico-legal recommendations to stay at home, closures of businesses, or banning of events and gatherings^5^.

As a result of social isolation imposed by some states and local authorities to avoid human-to-human transmission of the virus, there have been drastic reductions in daily physical activities routine and exercise. This has led to marked increase in sedentary behavior, with prolonged sitting, reclining, or lying down for screening activities (playing games, watching television, using mobile devices); minimized human movement and muscular activities (hence lower energy expenditure)^6^. Summarily, people tend to sit more and move less. A shift to a sedentary lifestyle during this pandemic is an issue of great concern because of its deleterious health implications.

The immune system is very responsive to exercise^4^. It is therefore imperative to keep the immune system well-primed because maladjusted and dysfunctional immune responses may increase the coronavirus disease progression to severe stages which may result in perturbed pulmonary gas exchange and eventual death.

There are two forms of immunological response: innate response and adaptive response. Macrophages, natural killer cells (NK), nitric oxide (NO) and other microbicide molecules constitute the cellular component of innate response. Chemical barriers (e.g. complement system) and physical barriers (e.g. skin) are also components of innate response. The adaptive immune response can be divided into humoral responses mediated by cytokines and antibodies produced by B-lymphocytes and cellular response mediated by T-lymphocytes (CD4+ and CD8)^7^. Many of the factors that guide an immunological response and determine whether an infection is controlled, or not can be modulated by exercise^4^.

### The need for regular exercise among older People

Exercise is a physical activity that is planned, well structured, and repetitive with the aim of improving or maintaining physical fitness^8^. Aging is linked with impairment in the modulation of the immune system known as immunosenescence^9^. Innate and adaptive immunity undergo cellular and molecular alterations as aging progresses, leading to heightened occurrences of infectious disease morbidity and mortality as well as increased deleterious inflammatory conditions^4^,^9^.

Exercise, as a therapy, has been shown to be very effective in attenuating the processes of immunosenescence. The advantages of exercise over other forms of therapy are manifold: low-cost, non-invasive, easy to implement, and amenable to being practiced in either home or clinical settings^4^,^10^. Regular exercise by older people can bring about a reduction in the incidence or severity of infectious disease. Though this is still being investigated with limited data, a number of reports from epidemiological studies and clinical exercise intervention trials supports this possibility. The effects of exercise impact multiple aspects of immune response including T cell phenotype and proliferation, antibody response to vaccination, and cytokine production^7^. In a study that included older and younger women, the risk of community-acquired pneumonia decreased with increasing physical activity^11^. In a related study, women aged 55 to 80 were followed for a period of 6 years, and physical inactivity in this age group was observed to be associated with increased risk of hospitalization due to infection^12^. Studies have also shown that exercise increases antibody response to influenza vaccine two weeks post-immunization^13^. Some epidemiological studies in older adults have also reported that greater levels of physical activity were associated with lower serum levels of several inflammatory markers including IL-6, TNF-α, and CRP^14^,^15^. However, although elevated inflammatory markers have been associated with poorer health outcomes, inflammation remains an essential component of immune defense^4^.

The benefits of exercise is not just confined to immunity. There are several other benefits that encompass both physical and psychosocial aspects. At the physical realm, exercise have been shown to improve mobility and motor control in older adults^16^ (Whitehurst et al., 2005), reducing rates of falls or other ambulation-associated injuries^17^. Exercise improves functional outcomes for disease states such as osteoarthritis^18^ and may positively impact on cancer treatment and recovery^19^.

At the psychosocial level, there exist a strong link between exercise programs and increases in reported vitality and decreased visits to doctor's offices^16^. Reduced depression, anxiety, and increased sense of coherence are all associated with constant exercise^20^,^21^. These evidences lend credence to the fact that long-term, moderate physical activity interventions in geriatric populations have several benefits including reduction in infectious disease risk, increased rates of vaccine efficacy, and improvements in both physical and psychosocial aspects of daily living.

### Myokines during exercise

The discovery that contracting skeletal muscle stimulates the production, secretion and expression of cytokines known as myokines, has deepened the understanding on how exercise induced-myokines modulate metabolic processes through autocrine, paracrine or endocrine mechanism^22^,^23^. Some identified myokines include interleukin-15 (IL-15), brain-derived neurotrophic factor (BDNF), leukemia inhibitory factor (LIF), irisin, fibroblast growth factor 21 (FGF-21), interleukin-6 (IL-6), interleukin-10 (IL-10) and secreted protein acidic and rich in cysteine (SPARC)^23^,^24^.

The most well-known exercise-induced myokine interleukin (IL)-6 was the first myokine to be identified in the bloodstream in response to muscle contractions^24^. IL-6 is a peptide that plays an anti-inflammatory role by inhibiting tumor necrosis factor; it also improves glucose uptake by stimulating AMP-activated protein kinase (AMPK) signaling. Surprisingly, circulating IL-6 levels are increased during exercise without any sign of muscle damage. It appears that, unlike IL-6 signaling in macrophages which leads to an inflammatory response, muscle cells produce and release IL-6 without activating classical pro-inflammatory pathways^24^,^25^. The fact that IL-6 can sometimes act as a pro-inflammatory and sometimes as an anti-inflammatory agent appears to be more dependent on whether it is expressed by muscle cells or by immune cells (macrophages)^26^.

BDNF is a receptor tropomyosin-related kinase extensively expressed in the brain. It enhances glucose utilization, controls body composition and energy homeostasis through the hypothalamic pathway, and regulates metabolism in skeletal muscle. BDNF appears to be a myokine that acts in an autocrine or paracrine fashion with strong effects on peripheral metabolism, including fat oxidation, and a subsequent effect on the size of adipose tissue^23^.

IL-15 may play a role not only in muscle-fat interaction, but also in skeletal muscle fiber growth. Among its various roles, IL-15 has been demonstrated to regulate metabolic diseases, such as obesity and diabetes^23^. LIF is produced by skeletal muscle and affects intact muscles, as well as isolated muscle cells. Among its various roles, the most important role of LIF in muscle satellite cell is proliferation for proper muscle hypertrophy and regeneration^23^.

Findings from previous studies indicated that exercise-induced myokines might be the potential candidates to provide beneficial effects by stimulating metabolic pathways, improving glucose uptake, improving fat oxidation, and regulating skeletal muscle regeneration. Just as exercise seems to promote the myokine response, physical inactivity seems to impair it, and could be a mechanism to explain the association between sedentary behavior and many chronic diseases^23^–^24^, ^27^.

### Exercise promotes an environment of anti-inflammatory cytokines

Adipokines are identified as hormones that mediate crosstalk between adipose tissue and brain, as well as metabolic functions during the activation of tissues^23^. The proinflammatory role of various adipocyte-produced adipokines has also been identified^23^. Tumor necrosis factor, chemokine C-C motif ligand-2, and plasminogen activator inhibitor-1 are proinflammatory adipokines that are overly secreted in obesity, leading to metabolic and cardiovascular diseases. Proinflammatory effects of these adipokines have now been clearly recognized to be counterbalanced by the protective effects of skeletal muscle-secreted peptides. Exercise-induced benefits are well known to prevent harmful effects of proinflammatory adipokines through skeletal muscle-secreted proteins^23^.

A number of papers have documented that self-reported physical activity or physical performance is correlated inversely with systemic low-level inflammation, suggesting that the anti-inflammatory activity induced by regular exercise may exert some of the beneficial health effects of exercise in patients with chronic diseases^28^. The fact that the classical pro-inflammatory cytokines, TNF-α and IL-1 in general do not increase with exercise, whereas exercise provokes an increase in circulating levels of well-known anti-inflammatory cytokines and cytokine inhibitors such as IL-1ra, IL-10 and sTNF-R suggests that exercise provokes an environment of anti-inflammatory cytokines^29^.

## Conclusion

Small changes induced by exercise satisfy essential requirements for a healthy life. Exercise is also an appealing therapy from a logistical vantage point, as it is relatively easy to implement, requires little cost or equipment, and can be performed in either clinical or home settings. This will help to keep the immune system in tiptop shape and increase the functional outcome in combating infectious diseases. To obtain the best results, practice of physical exercise should be regular. In addition, given the role of myokines in fine-tuning some metabolic processes, exercise provide a molecular explanation for the extensive cross-talk between muscle and other tissues in our body and reveal novel therapeutic avenues for the treatment of a myriad of chronic diseases that are associated with a sedentary life-style. While the scientific community is working to provide vaccines and treatments that will eradicate the novel coronavirus (COVID-19), the need for strengthening the immune system, especially among the geriatric population cannot be overemphasized.

## References

[1] World Health Organization Infection prevention and control during health care when COVID-19 is suspected.

[2] Adhikari SP, Meng S, Wu YJ, Mao YP, Ye RX, Wang QZ, Sun C, Sylvia S, Rozelle S, Raat H, Zhou H (2020). Epidemiology, causes, clinical manifestation and diagnosis, prevention and control of coronavirus disease (COVID-19) during the early outbreak period: a scoping review. Infectious Diseases of Poverty.

[3] Li G, Fan Y, Lai Y, Han T, Li Z, Zhou P, Pan P, Wang W, Hu D, Liu X, Zhang Q (2020). Coronavirus infections and immune responses. Journal of Medical Virology.

[4] Nieman DC, Wentz LM (2019). The compelling link between physical activity and the body's defense system. Journal of Sport and Health Science.

[5] Centers for Disease Control and Prevention (2020). How it spreads.

[6] Inyang DM, Stella OO (2015). Sedentary lifestyle: health implications. IOSR Journal of Nursing and Health Science.

[7] Rodrigo T, Silvia AC, Veronica SP, Patricia ML (2012). Effect of exercise on the immune sytem: response, adapation and cell signaling. Exercise and Sport Science.

[8] Langhammer B, Bergland A, Rydwik E (2018). The importance of physical activity exercise among older people. BioMed research international.

[9] Senchina DS, Kohut ML (2007). Immunological outcomes of exercise in older adults. Clinical Interventions in Aging.

[10] Kohut ML, Senchina DS (2004). Reversing age-associated immunosenescence via exercise. Exerc Immunol Rev.

[11] Baik I, Curhan GC, Rimm EB, Bendich A, Willett WC, Fawzi WW (2000). A prospective study of age and lifestyle factors in relation to community-acquired pneumonia in US men and women. Archives of Internal Medicine.

[12] Leveille SG, Resnick HE, Balfour J (2000). Gender differences in disability: evidence and underlying reasons. Aging Clinical and Experimental Research.

[13] Kohut ML, Senchina DS, Madden KS, Martin AE, Felten DL, Moynihan JA (2004). Age effects on macrophage function vary by tissue site, nature of stimulant, and exercise behavior. Experimental Gerontology.

[14] Taaffe DR, Harris TB, Ferrucci L, Rowe J, Seeman TE (2000). Cross-sectional and prospective relationships of interleukin-6 and C-reactive protein with physical performance in elderly persons: MacArthur studies of successful aging. The Journals of Gerontology Series A: Biological Sciences and Medical Sciences.

[15] Colbert LH, Visser M, Simonsick EM, Tracy RP, Newman AB, Kritchevsky SB, Pahor M, Taaffe DR, Brach J, Rubin S, Harris TB (2004). Physical activity, exercise, and inflammatory markers in older adults: findings from the Health, Aging and Body Composition Study. Journal of the American Geriatrics Society.

[16] Whitehurst MA, Johnson BL, Parker CM, Brown LE, Ford AM (2005). The benefits of a functional exercise circuit for older adults. Journal of Strength and Conditioning Research.

[17] Schoenfelder DP, Rubenstein LM (2004). An exercise program to improve fall-related outcomes in elderly nursing home residents. Applied Nursing Research.

[18] Foy CG, Wickley KL, Adair N, Lang W, Miller ME, Rejeski WJ, Woodard CM, Berry MJ (2006). The Reconditioning Exercise and Chronic Obstructive Pulmonary Disease Trial II (REACT II): Rationale and study design for a clinical trial of physical activity among individuals with chronic obstructive pulmonary disease. Contemporary Clinical Trials.

[19] Galvão DA, Newton RU (2005). Review of exercise intervention studies in cancer patients. Journal of Clinical Oncology.

[20] Antunes HK, Stella SG, Santos RF, Bueno OF, Mello MT (2005). Depression, anxiety and quality of life scores in seniors after an endurance exercise program. Brazilian Journal of Psychiatry.

[21] Kohut ML, Arntson BA, Lee W, Rozeboom K, Yoon KJ, Cunnick JE, McElhaney J (2005). Moderate exercise improves antibody response to influenza immunization in older adults. Vaccine.

[22] Pedersen BK, Akerstrom TC, Nielsen AR, Fischer CP (2007). Role of myokines in exercise and metabolism. Journal of Applied Physiology.

[23] So B, Kim HJ, Kim J, Song W (2014). Exercise-induced myokines in health and metabolic diseases. Integrative Medicine Research.

[24] Pedersen BK (2011). Muscles and their myokines. Journal of Experimental Biology.

[25] Plomgaard P, Penkowa M, Pedersen BK (2005). Fiber type specific expression of TNF-alpha, IL-6 and IL-18 in human skeletal muscles. Exerc Immunol Rev.

[26] Pedersen BK, Febbraio MA (2008). Muscle as an endocrine organ: focus on muscle-derived interleukin-6. Physiological Reviews.

[27] Schnyder S, Handschin C (2015). Skeletal muscle as an endocrine organ: PGC-1α, myokines and exercise. Bone.

[28] Petersen A, Pedersen B (2006). The role of IL-6 in mediating the anti inflammatory. J Physiol Pharmacol.

[29] Ostrowski K, Schjerling P, Pedersen BK (2000). Physical activity and plasma interleukin-6 in humans-effect of intensity of exercise. European Journal of Applied Physiology.

